# Pan-cancer analysis identifies LMNB1 as a target to redress Th1/Th2 imbalance and enhance PARP inhibitor response in human cancers

**DOI:** 10.1186/s12935-022-02467-4

**Published:** 2022-03-03

**Authors:** Haixiang Qin, Yingqiang Lu, Lin Du, Jingyan Shi, Haoli Yin, Bo Jiang, Wei Chen, Wenli Diao, Meng Ding, Wenmin Cao, Xuefeng Qiu, Xiaozhi Zhao, Hongqian Guo

**Affiliations:** Department of Urology, Drum Tower Hospital, Medical School of Nanjing University, Institute of Urology, Nanjing University, 321 Zhongshan Road, Nanjing, 210008 Jiangsu People’s Republic of China

**Keywords:** LMNB1, Pan-cancer, Survival, Immune infiltration, Homologous recombination repair

## Abstract

**Background:**

Emerging evidence suggests that LMNB1 is involved in the development of multiple cancer types. However, there is no study reporting the potential role of LMNB1 in a systematic pan-cancer manner.

**Methods:**

The gene expression level and potential oncogenic roles of LMNB1 in The Cancer Genome Atlas (TCGA) database were analyzed with Tumor Immune Estimation Resource version 2 (TIMER2.0), Gene Expression Profiling Interactive Analysis version 2 (GEPIA2), UALCAN and Sangerbox tools. Pathway enrichment analysis was carried out to explore the possible mechanism of LMNB1 on tumorigenesis and tumor progression. The therapeutic effects of LMNB1 knockdown combined with PARP inhibition on human cancers were further investigated in vitro.

**Results:**

LMNB1 upregulation is generally observed in the tumor tissues of most TCGA cancer types, and is verified in kidney renal clear cell carcinoma using clinical specimens of our institute. High level of LMNB1 expression usually predicts poor overall survival and disease free survival for patients with tumors. Mechanically, LMNB1 level is positively correlated with CD4+ Th2 cell infiltration and DNA homologous recombination repair gene expression. In vitro experiments reveal that targeting LMNB1 has a synergistic effect on prostate cancer with PARP inhibitor treatment.

**Conclusions:**

LMNB1 is a biomarker of CD4+ Th2 cell infiltration and DNA homologous recombination repair in human cancers. Blockage of LMNB1 combined with PARP inhibitor treatment could be a promising therapeutic strategy for patients with cancers.

**Supplementary Information:**

The online version contains supplementary material available at 10.1186/s12935-022-02467-4.

## Background

Cancer is a complicated disease caused and influenced by genetic risks and environment factors. It is generally recognized that adverse genetic changes such as p53 gene mutations are significant risk factors associated with nearly all kinds of human tumors. With the bioinformatic advancements based on public cancer datasets and repositories, such as The Cancer Genome Atlas (TCGA), Gene Expression Omnibus (GEO) and Clinical Proteomic Tumor Analysis Consortium (CPTAC), pan-cancer analysis of interested oncogenes or tumor suppressive genes can be performed to explore the universal mechanisms of tumorigenesis and provide potential targets of general strategies for cancer therapy.

Lamin B1 is one of the two B-type lamin proteins [1, 2], which is encoded by LMNB1 gene (NCBI Entrez Gene: 4001). Lamin proteins are evolutionarily conserved among vertebrates [3], and located between the inner nuclear membrane and the peripheral heterochromatin, playing important roles in making up nuclear envelope and maintaining nuclear stability [4]. Lamins are found implicated in the regulation of in a wide variety of nuclear processes, including DNA replication and repair, mitotic spindle assembly, chromosome distribution, oxidative stress response, and gene expression [5–10]. Recently, increased studies have revealed that LMNB1 is abnormally expressed in a lot of human cancer types, such as upregulated in lung adenocarcinoma [11], prostate cancer [12, 13], cervical cancer [14], pancreatic cancer [15], liver cancer [16], and downregulated in breast cancer [17], gastric cancer [18] and two subtypes of lung cancer [19]. Though previous evidences showed that LMNB1 expression affected the clinical behavior and patient outcome of the above cancer types, the precise mechanism underlying carcinogenesis of LMNB1 has not been extensively explored.

Hence, this pan-cancer analysis was designed to investigate the precise role and possible mechanism of LMNB1 in all human cancer types. In brief, we comprehensively analyzed the mRNA and protein levels of LMNB1, and its association with prognostic landscape across all TCGA cancer types. Considering that malignant tumors often show a unique immunosuppressive microenvironment mediated by varieties of inflammation-related stromal cells and tumor-infiltrating immune cells, which results in poor response to immunotherapy and worse prognosis [20–23], and previous research indicated a significant correlation between LMNB1 expression and several kinds of tumor infiltrating lymphocytes in renal cell carcinoma [24], so we further investigated whether LMNB1 is involved in immune signaling pathways in a systematic pan-cancer manner, aiming to identify novel therapeutic strategy. Moreover, pathway enrichment analysis led us to focus attention on the relevance between LMNB1 expression and DNA homologous recombination repair (HRR), which plays a critical role in DNA repair and genome integrity. Loss of function alterations in HRR-associated genes such BRCA1 and BRCA2, resulted in better response to poly ADP-ribose polymerase (PARP) inhibitor (PARPi) treatment in cancer patients [25, 26]. We also investigated the effects of LMNB1 knockdown on PARPi treatment response in human prostate cancer.

To the best of our knowledge, this is the first pan-cancer analysis of LMNB1 gene, which showed that LMNB1 is not only a biomarker of CD4+ Th2 cell infiltration and DNA homologous recombination repair, but also a promising therapeutic target to redress Th1/Th2 imbalance and enhance PARPi response.

## Materials and methods

### Cell lines and cell culture

Human immortalized normal prostate epithelial cell line RWPE-1 and human prostate cancer cell line C4-2 were purchased from the American Type Culture Collect (Manassas, USA). Human benign prostate hyperplasia cell line BPH-1 were obtained from the Leibniz Institute DSMZ (Braunschweig, Germany). Other human prostate cancer cell lines namely LNCaP, VCaP, 22Rv1, DU145 and PC3 were purchased from the Cell Bank of Type Culture Collection, Chinese Academy of Science (Shanghai, China). RWPE-1, LNCaP, 22Rv1, DU145 and PC3 cell lines have been authenticated using STR profiling within the last 3 years. RWPE-1 cells were maintained in Keratinocyte Serum Free Medium (K-SFM, Gibco, 17005042, Grand Island, NY, USA) supplemented with 0.05 mg/ml bovine pituitary extract (Gibco, 17005042) and 5 ng/ml human recombinant EGF (Gibco, 17005042). BPH-1, LNCaP, C4-2 and 22Rv1 cells were maintained in RPMI-1640 (Gibco, 11875093) supplemented with 10% fetal bovine serum (FBS, Gibco, 10100147). VCaP and DU145 cells were maintained in DMEM (Gibco, 11995065) supplemented with 10% FBS. PC3 cells were maintained in F12K (Gibco, 21127022) supplemented with 10% FBS. All complete medium were supplemented with 100 units/ml penicillin and 100 μg/ml streptomycin. All cell experiments were performed with mycoplasma-free cells.

### Overexpression/shRNA lentivirus of LMNB1 and transduction

Overexpression and shRNA lentivirus of human LMNB1 were designed and provided by GenePharma (Shanghai, China). PC3 cells were transduced with the overexpression lentivirus (multiplicity of infection (MOI): 50) to establish LMNB1 stably overexpressing cells, while 22Rv1 cells were transduced with the shRNA lentivirus (MOI: 10) to acquire LMNB1 stably knockdown cells. The transformed cells were selected by 1 μg/ml puromycin (Selleck, S7417, Shanghai, China) for 3 days. Overexpression or knockdown efficiency was determined by qRT-PCR.

### Cell viability assay

6 × 10^3^ cells suspended in 100 μl complete medium were seeded in 96-well plates per well. 12 h later, olaparib (Selleck, S1060) with a series of concentrations was added to the medium. After incubating at 37 ℃ for 72 h, the viability of prostate cancer cells was evaluated with Cell Counting Kit 8 (CCK-8) (Vazyme Biotech, A311, Nanjing, China) and detected at OD450 nm with a microplate reader (TECAN Group Ltd., infinite M200pro, Männedorf, CH/CHE). The half maximal inhibitory concentration (IC_50_) was acquired with the cell viability assay.

### Patients and clinical specimens

This study was approved by the Ethics Committee of Drum Tower Hospital, Medical School of Nanjing University (Nanjing, China) and conducted in accordance with the Declaration of Helsinki principles. Clinical renal and prostate specimens of radical surgery along with corresponding clinicopathological characteristics were collected from June 2011 to June 2016. Neither chemotherapy nor radiotherapy was performed to the patients before surgery. In total, 25 pairs of frozen KIRC tissues and corresponding adjacent normal tissues were prepared for qRT-PCR analysis, and nine pairs for western blotting experiment. 130 formalin-fixated KIPC tissues of which 46 cases were accompanied by corresponding noncancerous kidney tissues, and 81 formalin-fixated PRAD tissues were collected for immunohistochemistry staining.

### RNA isolation and quantitative real-time PCR (qRT-PCR)

Total RNA of cancer cells or surgical specimens was extracted using TRIzol^®^ reagent (Invitrogen, 15596018, Carlsbad, CA, USA) and 1 μg total RNA was reversed to complementary DNA in 20 μl reaction system with PrimeScript™ RT Master Mix kit (TaKaRa Biotechnology (Dalian), RR036A, Dalian, China). qRT-PCR was performed by using ChamQ Universal SYBR qPCR Master Mix (Vazyme Biotech, Q711) with the QuantStudio™ 6 Flex Real-Time PCR System (Applied Biosystems, 4485692, Foster City, CA, USA). The primer concentration used for the qRT-PCR is 10 μM. Relative mRNA levels of target genes were normalized to ACTB by the 2^−△△CT^ algorithm. Specially, when qRT-PCR was performed with clinical samples, both ACTB and GAPDH were set as endogenous control to avoid inaccurate interruption of results. Every qRT-PCR experiment was performed in triplicate and repeated three times independently. The primer sequences used in the study were listed in Additional file 2: Table S1.

### Western blotting

Protein extraction and western blotting were performed as described previously [27]. 20 μg total proteins in the lysates were loaded in and separated by SDS-PAGE. Primary antibodies for lamin B1 (1:5000, abcam, ab133741, Cambridge, MA, USA), cleaved PARP (Asp214) (1:1000, Cell Signaling Technology (CST), #5625, Danvers, MA, USA), cleaved caspase-3 (Asp175) (1:1000, CST, #9661), β-actin (1:1000, CST,#4970) and GAPDH (1:1000, CST, #5174) were used to bind the corresponding targets. After incubating with horseradish peroxidase-conjugated secondary antibodies, the protein bands were visualized with standard chemical luminescence methodology.

### Immunohistochemistry (IHC)

IHC staining was carried out on 3 µm thick paraffin-embedded sections with primary antibodies against lamin B1 (1:3000, abcam, ab133741) and BRCA1 (1:1000, abcam, ab16780). All the sections of IHC staining were scored by two well-trained pathologists independently. The staining intensity was scored as 0 (negative), 1 (weak), 2 (moderate), and 3 (strong). The staining range in the tumor area was scored as 0 (0%), 1 (1–25%), 2 (26–50%), 3 (51–75%) and 4 (75–100%). The final score was obtained by multiplying the intensity score and the staining range, which was defined as negative or low expression when ≤ 6 or as high expression when > 6. Five fields from each section were randomly chosen and evaluated.

### Gene and protein expression analysis

To determine the expression pattern of LMNB1 in healthy human beings, we explored The Human Protein Atlas (HPA) (https://www.proteinatlas.org/). Consensus normalized expression (NX) was obtained by combining the data of several sources, including HPA, The Genotype-Tissue Expression (GTEx), The Functional Annotation of Mammalian Genomes 5 (FANTOM5), Single Cell Expression Atlas, the Human Cell Atlas, GEO, and the European Genome-phenome Archive. The protein concentrations in blood were determined by mass spectrometry-based plasma proteomics and estimated from spectral counts in the Peptide Atlas. “Enhanced” was defined as NX levels of a group (of 1–5 tissues or 1–10 cell types) at least 4 times the mean of other tissue or cell types. “Low specificity” was defined as NX levels not less than 1 in at least one tissue or cell type but not elevated in any tissue or cell type.

Tumor Immune Estimation Resource version 2 (TIMER2.0) (http://timer.cistrome.org/) [28–30] and Gene Expression Profiling Interactive Analysis version 2 (GEPIA2) (http://gepia2.cancer-pku.cn/#index) [31] provided powerful assistance in studying the differential expression of LMNB1 in TCGA database. We employed “Gene_DE” module of TIMER2.0 to obtain the box-whisker plots of LMNB1 expression between tumor and adjacent normal tissues across all TCGA tumors, and “Stage Plot” module of GEPIA2 to acquire the violin plots of LMNB1 expression grouped in different pathological stages of the TCGA tumor types whose data of pathological stage characteristic were available. The log_2_TPM transformed expression data were used for plotting.

Differential protein expression of LMNB1 was analyzed with UALCAN (http://ualcan.path.uab.edu/index.html) [32], which is a comprehensive webserver providing protein expression analysis based on the data from CPTAC [33].

### Gene correlation analysis

We computed the correlation of LMNB1 and other genes or signatures of interest in the “Correlation Analysis” pane of GEPIA2. According to the user tutorial of GEPIA2, the non-log scale was used for calculation and the log-scale axis for visualization, and the mean value of the log_2_TPM of the genes included in a signature was used as the signature score [31]. The heatmap of correlation between LMNB1 gene and target signature across all TCGA tumors was drawn by R language with “heatmap” package. Furthermore, we used “Gene_Corr” module of TIMER2.0 to get a detailed heatmap of the correlation between LMNB1 and every single gene in the specific signature in various TCGA cancer types.

### Survival analysis

We performed survival analysis with GEPIA2 tools. Heatmap of overall survival (OS) and disease free survival (DFS) based on LMNB1 expression across all TCGA tumor types were obtained in the “Survival Map” module, then Kaplan–Meier curves of the tumor types with logrank *p* value < 0.05 were drawn in the “Survival analysis” module. The group cutoff was set as 50% of LMNB1 expression.

### Immune infiltration analysis

Immune pathways analysis was performed using the Sangerbox tools, a free online platform for TCGA data analysis (http://www.sangerbox.com/tool). Then we employed the “Immune-Gene” module of TIMER2.0 to investigate the details of immune cell infiltration levels of the selected immune pathways (e.g. CD4+ T cells) in a variety of TCGA tumors. The results were displayed in a heatmap with colors indicating the purity-adjusted Spearman’s rho coefficient across all TCGA cancers, and in scatter plots showing the relationship between LMNB1 expression and immune infiltration estimation with detailed Spearman’s rho coefficient and *p* value of interested cancer types.

### Function enrichment analysis

In the “Similar Genes Detection” pane of GEPIA2, we obtained the top 100 genes that presented a similar expression pattern with LMNB1 in the combined data of all the TCGA tumor tissues. On the other hand, we acquired another top 50 genes whose corresponding proteins were capable of binding to lamin B1 in STRING website (https://string-db.org/). The parameter of active interaction sources was set as “experiments”, minimum required interaction score as “low confidence (0.150)”, max number of interactors to show as “no more than 50 interactors (in 1st shell)”, and the other checkboxes were kept default. We combined the two screened gene lists to performed Kyoto encyclopedia of genes and genomes (KEGG) and Gene ontology (GO) enrichment analysis. Briefly, the above gene aggregate was uploaded to Database for Annotation, Visualization, and Integrated Discovery (DAVID) website (https://david.ncifcrf.gov/) to acquire the enriched pathways associated with LMNB1 gene in homo sapiens, which were then visualized by R language with “ggplot2” package. GO term enrichment analysis was also conducted in R language with “clusterProfiler” package.

Gene Set Enrichment Analysis (GSEA) of TCGA datasets was performed with the “GSEA4.0” software (http://www.gsea-msigdb.org/gsea/index.jsp) according to the manufacture’s instruction [34, 35]. The mRNA expression data of TCGA cohort which was downloaded using Sangerbox tools was uploaded to the software, then analyzed with the “h.all.v7.4.symbols.gmt [Hallmarks]”, “c2.cp.kegg.v7.4.symbols.gmt [Curated]” and “c5.go.bp.v7.4.symbols.gmt [Gene ontology]” gene sets of the Molecular Signatures Database (MSigDB), by using LMNB1 gene expression as the phenotype.

### Statistical analysis

R programming language (version 4.0.4), IBM SPSS Statistics (Version 22.0) and GraphPad Prism (version 8.0.2) were used for statistical analysis. Continuous normally distributed variables were presented as mean ± standard deviation, and compared using unpaired Student’s t test. Categorical variables were analyzed by Chi-square test or Fisher exact test. Pearson and Spearman correlation analyze was employed to evaluate the correlation between two continuous normally distributed factors or categorical variables, respectively. *p* < 0.05 was considered statistically significant.

## Results

### LMNB1 upregulation was observed in various cancer types and validated in kidney renal clear cell carcinoma

The expression and distribution of LMNB1 in different human tissues and cell types under physiological conditions was explored in the HPA website. LMNB1 was widely expressed in almost all the tissue types while especially enhanced in the lymphoid tissue, including thymus, appendix, lymph node, tonsil and bone marrow (Additional file 1: Fig. S1A). Based on single cell RNAseq of human issues and blood cells, LMNB1 showed highest expression in granulocytes, monocytes, spermatids and extravillous trophoblasts (Additional file 1: Fig. S1B), whereas low expression specificity in blood cell types (Additional file 1: Fig. S1C). Based on the mass spectrometry of human plasma in the publicly available Peptide Atlas, the protein concentration of lamin B1 was estimated as 3.3 μg/l (Additional file 1: Fig. S1D).

**Fig. 1:**
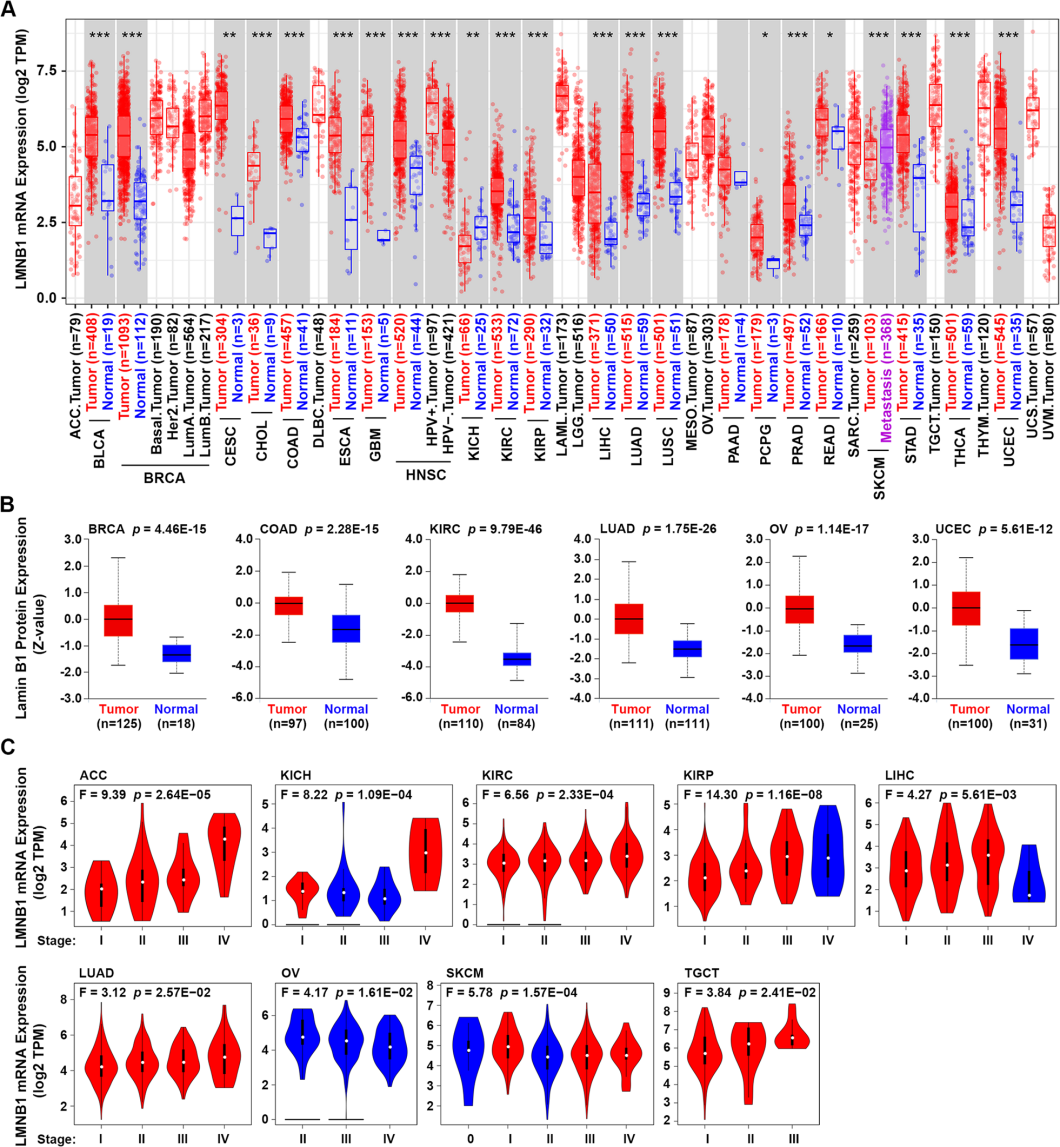
mRNA and protein expression of LMNB1 in human cancers. **A** LMNB1 mRNA expression in diverse human cancers or cancer subtypes was visualized by TIMER2.0 based on TCGA database. The statistical significance was calculated with the Wilcoxon test (*p < 0.05; **p < 0.01; ***p < 0.001). **B** Total protein level of lamin B1 in the tumor tissues and corresponding normal tissues of BRCA, COAD, KIRC, LUAD, OV and UCEC in the CPTAC database was compared using UALCAN. Significance of difference was evaluated by Student’s t test. **C** mRNA expression of LMNB1 was analyzed in different pathological stages in TCGA cancers. One-way ANOVA was performed to estimate the significance of LMNB1 expression in different groups

The TIMER2.0 webserver was applied to study the differential expression of LMNB1 between tumor and adjacent normal tissues across 33 human cancer types in TCGA database. As shown in Fig. 1A, in the 21 distinct tumor types of which normal tissue data are available, LMNB1 expression in the tumor tissues is upregulated compared to the corresponding adjacent tissues in 19 kinds of tumors, including BLCA (Bladder Urothelial Carcinoma), BRCA (Breast invasive carcinoma), CESC (Cervical squamous cell carcinoma and endocervical adenocarcinoma), CHOL (Cholangio carcinoma), COAD (Colon adenocarcinoma), ESCA (Esophageal carcinoma), GBM (Glioblastoma multiforme), HNSC (Head and Neck squamous cell carcinoma), KIRC (Kidney renal clear cell carcinoma), KIRP (Kidney renal papillary cell carcinoma), LIHC (Liver hepatocellular carcinoma), LUAD (Lung adenocarcinoma), LUSC (Lung squamous cell carcinoma), PCPG (Pheochromocytoma and Paraganglioma), PRAD (Prostate adenocarcinoma), READ (Rectum adenocarcinoma), STAD (Stomach adenocarcinoma), THCA (Thyroid carcinoma), UCEC (Uterine Corpus Endometrial Carcinoma) (Fig. 1A). In contrast, only in KICH (Kidney Chromophobe), LMNB expression in the tumor tissues is downregulated compared to the corresponding control tissues. Besides, LMNB1 expression in the metastasis tissues of SKCM (Skin Cutaneous Melanoma) is much higher than the primary tumor tissues. In addition to transcriptome analysis, we further compared the protein level of lamin B1 in different tumor kinds through UALCAN resource which provides protein expression analysis using data from CPTAC. The total protein expression of lamin B1 was found significantly higher in the tumor tissues of all the six tumor kinds (BRCA, COAD, KIRC, LUAD, OV (Ovarian serous cystadenocarcinoma) and UCEC) whose normal tissue data were available in CPTAC, than the corresponding normal tissues (Fig. 1B). Furthermore, we applied GEPIA2 approach to explore the relationship between LMNB1 mRNA expression and the pathological stages across all TCGA tumors. The results indicated that when the pathological stage increased, LMNB1 expression showed a trend of gradual increase in ACC (Adrenocortical carcinoma), KIRC, LUAD, TGCT (Testicular Germ Cell Tumors), and a trend of gradual decrease in OV, while no obvious gradual trend but with stage-specific expression difference in KICH, KIRP, LIHC and SKCM (Fig. 1C).

**Fig. 2:**
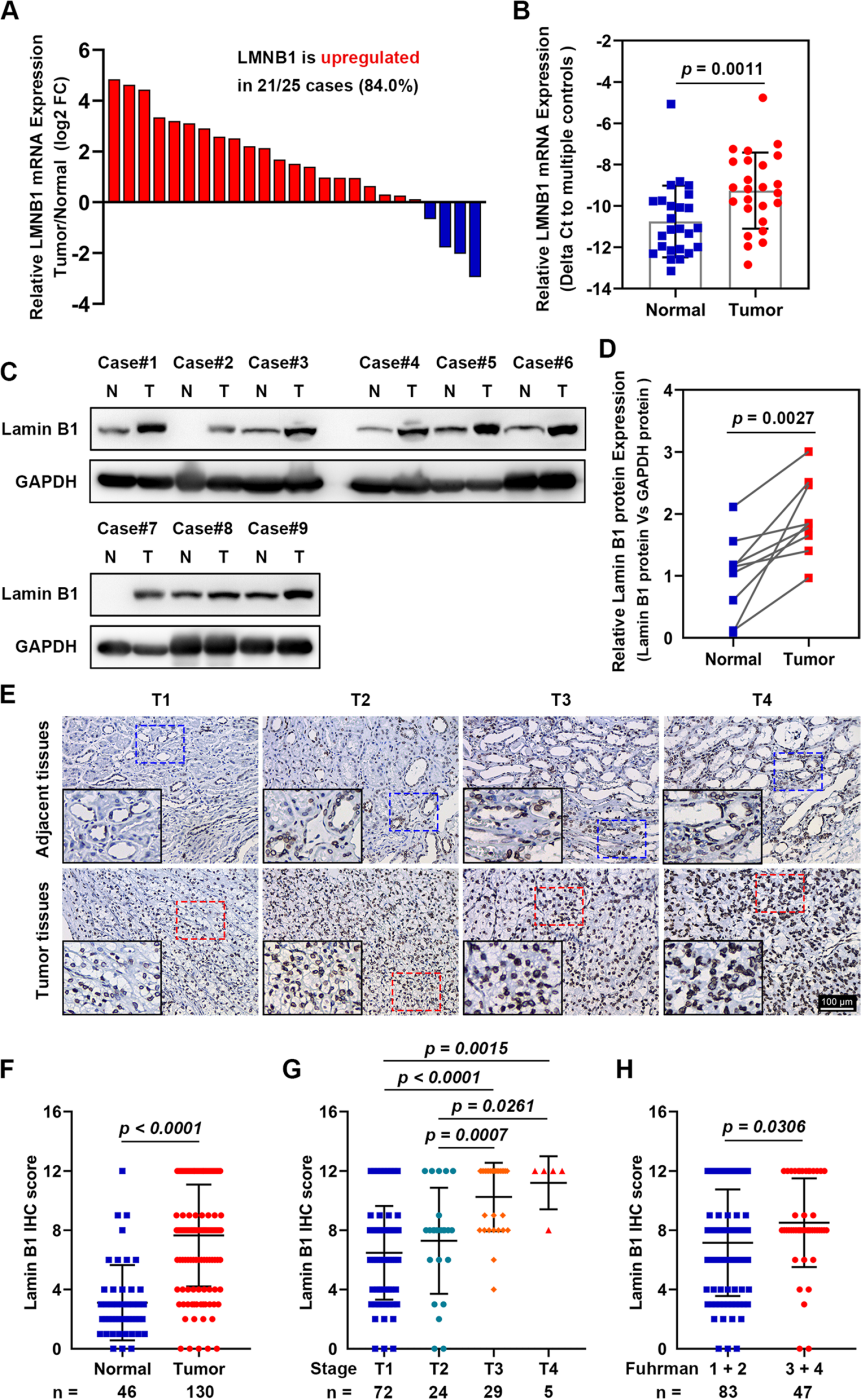
Validation of LMNB1 upregulation in KIRC clinical samples. **A** Relative LMNB1 mRNA expression was analyzed by qRT-PCR in 25 pairs of KIRC tumor tissues and adjacent normal tissues, with ACTB set as an internal control. **B** Significance of difference between tumor tissues and adjacent normal tissues was evaluated by Student’s t test. **C** Total protein level of lamin B1 in nine pairs of human KIRC specimens was detected by western blotting. **D** Quantitative analysis of LMNB1 protein levels was performed. **E** Representative images of lamin B1 IHC staining in normal renal tissues and KIRC specimens with different T stages (n = 130). Scale bars represent 100 μm. **F** IHC scores of lamin B1 in the normal areas and tumor tissues of renal. **G**, **H** Lamin B1 IHC scores in different T stages (**G**) and Fuhrman grades (**H**). Values were expressed as mean ± SD and analyzed by unpaired Student’s *t*-test between two groups

Since analysis of TCGA and CPTAC datasets showed that in KIRC and LUAD tumor tissues, LMNB1 expression was upregulated both in mRNA and protein levels, as well as increased gradually with elevated pathological stages, we decided to validate the LMNB1 expression status in specimens of radical nephrectomy in our center. Firstly, we detected LMNB1 mRNA expression in 25 pairs of KIRC tumor tissues and adjacent normal tissues by qRT-PCR. Compared to the corresponding non-cancerous tissues, 21 of 25 (84.0%) cancerous tissues showed higher LMNB1 mRNA expression (Fig. 2A, B). Furthermore, western blotting and IHC staining confirmed that lamin B1 protein level in tumor specimens was much higher than the corresponding normal tissues (Fig. 2C–F). Consistent with TCGA analysis, IHC staining showed that KIRC with higher T stage had higher expression of lamin B1 (Fig. 2F, G), and KIRC with advanced Fuhrman nuclear grade (G3 and G4) also showed higher lamin B1 expression than low grade (G1 and G2) (Fig. 2H). Moreover, we explored the correlation between the clinicopathological features and lamin B1 expression in surgical resected specimens from a cohort of 130 consecutive KIRC patients by IHC staining. The results showed that high lamin B1 IHC staining in KIRC patients was positively associated with male gender (*p* = 0.001), pathological T stage (*p* < 0.001), distant metastasis (*p* = 0.008), Fuhrman grade (*p* = 0.002) and microvascular invasion (*p* = 0.001) (Table 1). These results indicated that LMNB1 upregulation was generally correlated with tumor initiation and progression.

**Table 1:** The correlation of lamin B1 expression with clinicopathologic features in 130 KIRC patients

Clinicopathologic features	Lamin B1 IHC staining		*p* value
	Negative or low	High	
Gender			
Male	21 (44.7%)	61 (73.5%)	**0.001**
Female	26 (55.3%)	22 (26.5%)	
Age (year)			
≤ 50	16 (34.0%)	22 (26.5%)	0.364
> 50	31 (66.0%)	61 (73.5%)	
Pathological T stage			
T1	37 (78.7%)	35 (42.2%)	**< 0.001**
T2	8 (17.0%)	16 (19.3%)	
T3 + T4	2 (4.3%)	32 (38.5%)	
Lymph node involvement			
N0	46 (97.9%)	76 (91.6%)	0.257
N1	1 (2.1%)	7 (8.4%)	
Distant metastasis			
M0	45 (95.7%)	65 (78.3%)	**0.008**
M1	2 (4.3%)	18 (21.7%)	
Fuhrman grade			
1	12 (25.5%)	10 (12.0%)	**0.002**
2	27 (57.4%)	34 (41.0%)	
3 + 4	8 (17.1%)	39 (47.0%)	
Recurrence			
No	45 (95.7%)	76 (91.6%)	0.487
Yes	2 (4.3%)	7 (8.4%)	
Microvascular invasion			
No	44 (93.6%)	57 (68.7%)	**0.001**
Yes	3 (6.4%)	26 (31.3%)	

Statistical significance was calculated using the Chi-square test or the Fisher exact test

Bold value indicates a statistically significant difference

### LMNB1 upregulation was universally correlated with poor survival in human cancers

**Fig. 3:**
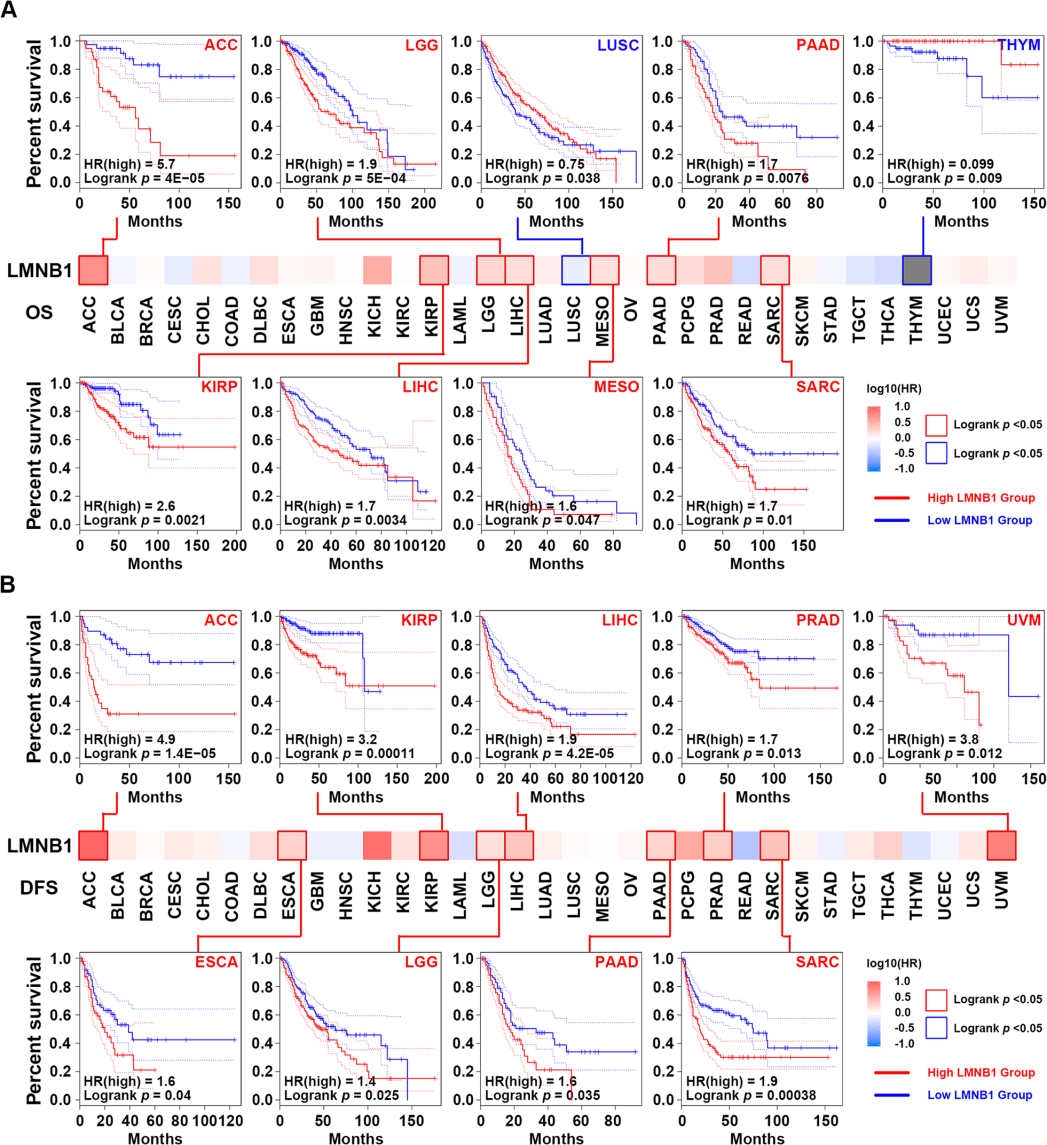
Relationship between LMNB1 mRNA expression and prognosis of TCGA cancers. **A**, **B** GEPIA2 was employed to get the survival heatmap of LMNB1 gene in TCGA datasets, and cancers with significant results were emphasized with solid line borders. The Kaplan–Meier curves with positive results of OS (**A**) and DFS (**B**) were displayed

Since high expression of LMNB1 was found in the overwhelming majority of cancer types and correlated with cancer aggressiveness, we wonder whether it affects the prognosis of human cancers. The “Survival analysis” module of GEPIA2 tool was applied to compare the OS and DFS based on the expression status of LMNB1 in TCGA datasets. As shown in Fig. 3A, LMNB1 expression higher than median was significantly associated with poor prognosis of OS for cancers of ACC (*p* < 0.0001), LGG (Brain Lower Grade Glioma) (*p* = 0.0005), PAAD (*p* = 0.0076), KIRP (*p* = 0.0021), LIHC (*p* = 0.0034), MESO (Mesothelioma) (*p* = 0.047), SARC (Sarcoma) (*p* = 0.01), while with favorable prognosis of OS for cancers of LUSC (*p* = 0.038), THYM (Thymoma) (*p* = 0.009) (Fig. 3A). On the other hand, DFS analysis revealed that highly expressed LMNB1 was notably correlated with poor prognosis for ACC (*p* < 0.0001), KIRP (*p* = 0.0001), LIHC (*p* < 0.0001), PRAD (*p* = 0.013), UVM (Uveal Melanoma) (*p* = 0.012), ESCA (*p* = 0.04), LGG (*p* = 0.025), PAAD (*p* = 0.035), SARC (*p* = 0.0004), while not correlated with favorable prognosis for any human cancer type (Fig. 3B).

### LMNB1 was identified as a biomarker of CD4+ Type 2 T helper cell infiltration in human cancers

**Fig. 4:**
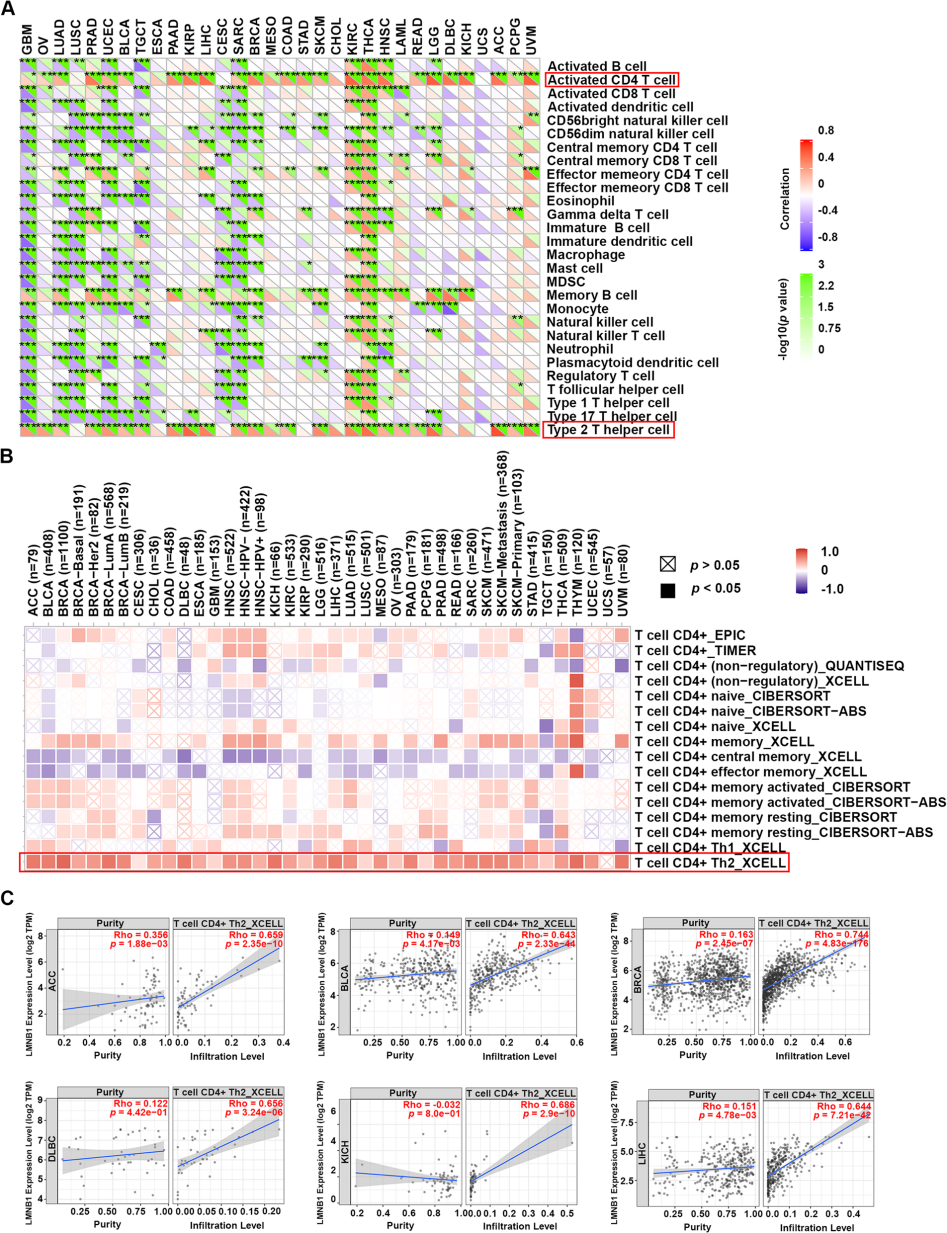
Correlation between LMNB1 mRNA expression and immune infiltration across all cancers in TCGA. **A** Immune pathway analysis was performed with sangerbox tool. The statistical significance was calculated with the Pearson test (*p < 0.05; **p < 0.01; ***p < 0.001). **B** The correlation between the mRNA expression of LMNB1 and the infiltration levels of CD4+ T cell subtypes across all TCGA tumors were analyzed with TIMER2.0. **C** The scatter plots of cancers with top purity-adjusted Spearman’s rho coefficient were showed

For the past few years, immune cells infiltrated in the tumor microenvironment (TME) has been found playing key roles in the tumorigenesis and tumor progression [36, 37]. Previous study has demonstrated that a gene hub including LMNB1 in KIRC was positively correlated with multiple kinds of tumor infiltrating lymphocytes, such as activated CD4+ T cells, CD8+ T cells, regulatory T cells and follicular helper T cells, but negatively correlated with resting mast cells, resting NK cells and activated NK cells [24]. Herein, we aimed to provide a comprehensive research on the potential relationship between LMNB1 expression and immune infiltration levels across all TCGA cancers. Firstly, Sangerbox tools helped us identified that activated CD4+ T cells and type 2 T helper cells were the main immune pathways which were significantly correlated with high expression of LMNB1 in most of human cancer types, especially ACC, KIRP, KIRC, LIHC, LGG, PRAD, THCA and UVM (Fig. 4A). Then we focus our attention on the details of CD4+ T cell infiltration levels. With the aid of TIMER2.0 resource, we employed the EPIC, TIMER, QUANTISEQ, XCELL, CIBERSORT, CIBERSORT-ABS methods to analyze the correlation between the infiltration levels of CD4+ T cell subtypes and the mRNA expression of LMNB1 across different TCGA tumor kinds. We found that all the TCGA cancers excluding UCS (Uterine Carcinosarcoma) showed statistical positive correlation between high LMNB1 expression and the immune infiltration of CD4+ Th2 cells based on XCELL algorithm (Fig. 4B). In contrast, the infiltration level of CD4+ central memory T cells and CD4+ effector memory T cells was found negatively correlated high LMNB1 expression in more than half of the TCGA cancer kinds (Fig. 4B). The scatter plots of the cancer types which occupied the top 10 purity-adjusted Spearman’s rho coefficient were showed (Fig. 4C, Additional file 1: Fig. S2). Since CD4+ Th2 cells and Th2 cytokines such as interleukin-4 (IL-4), IL-5, IL-6, IL10 and IL13 were thought to be associated with immunosuppressive contexture and tumor-promoting effects [38–43], we believed that LMNB1 could be considered as a biomarker of immunosuppressive microenvironment.

### Pathways of cell cycle and nuclear division were involved in the effects of LMNB1 on tumor pathogenesis

**Fig. 5:**
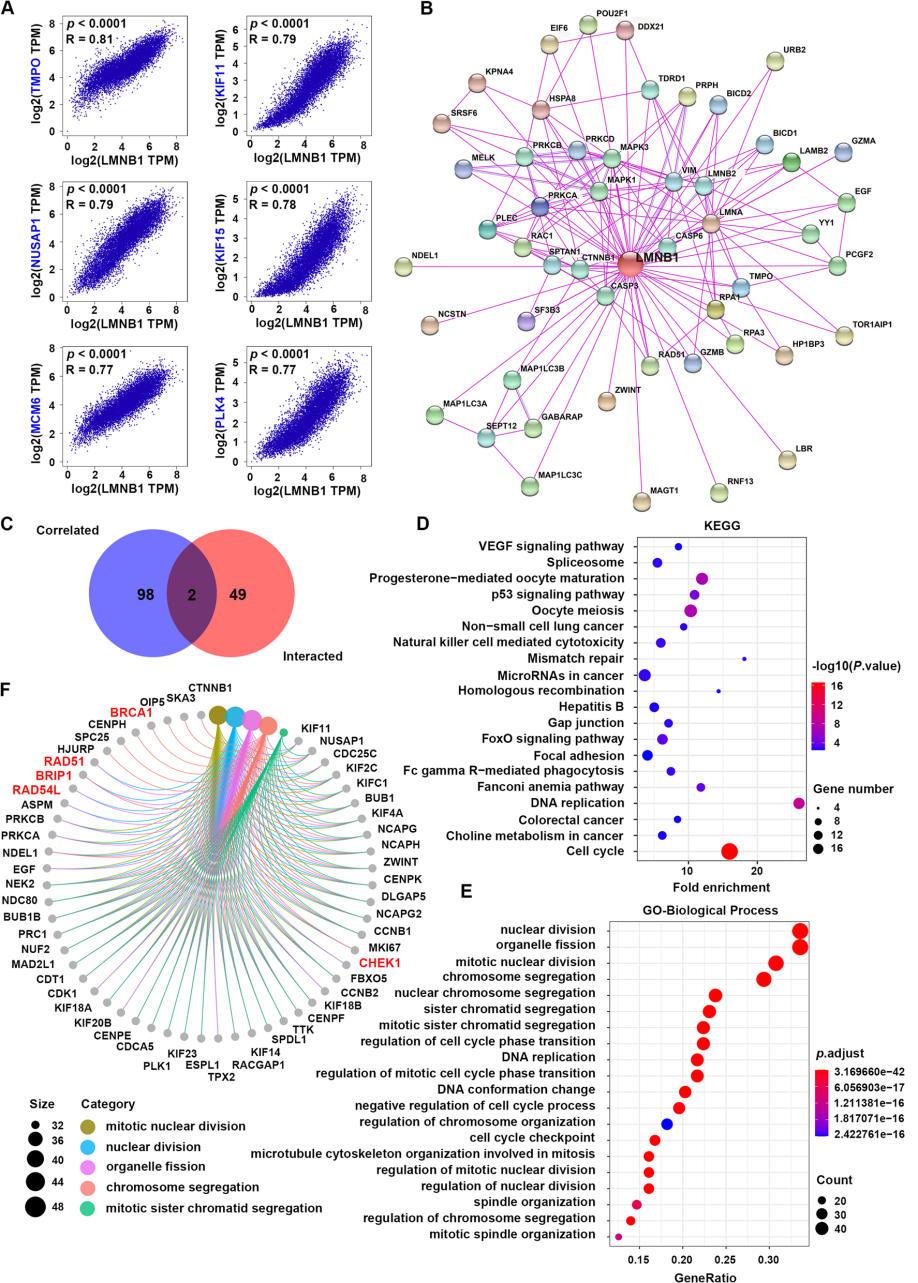
Function network of LMNB1 and LMNB1-related genes in TCGA. **A** GEPIA2 was used to acquire the top 100 LMNB1-correlated genes across all TCGA cancers. TMPO, KIF11, NUSAP1, KIF15, MCM6 and PLK4 were the most six correlated genes with LMNB1. **B** STRING network was used to obtain the top 50 genes of which the corresponding proteins have been experimentally determined lamin B1-binding. **C** The venn diagram visualized the intersection of LMNB1-correlated genes and lamin B1-binding proteins. **D** KEGG pathway analysis was conducted using LMNB1-correlated genes and lamin B1-binding proteins. **E** Biological process GO term enrichment analysis based on LMNB1-related partners was also performed. **F** Genes involved in the most enriched process were plotted

In order to further illustrate the molecular mechanism of LMNB1 in tumor initiation and progression, we screened out LMNB1-related genes for subsequent pathway enrichment analysis. By using GEPIA2 tool, we acquired the top 100 genes which had similar expression patterns with LMNB1 in the combined data of all TCGA cancer types, and the most six correlated genes are TMPO (thymopoietin) (R = 0.81), KIF11 (kinesin family member 11) (R = 0.79), NUSAP1 (nucleolar and spindle associated protein 1) (R = 0.79), KIF15 (kinesin family member 11) (R = 0.78), MCM6 (minichromosome maintenance complex component 6) (R = 0.77), PLK4 (R = 0.77) (Fig. 5A). The corresponding heatmap also verified that the above top six related genes showed statistical positive correlation with LMNB1 in almost every cancer type (Additional file 1: Fig. S3). On the other hand, with STRING website we obtained another top 50 genes which encode the proteins that had been experimentally proved to physically bind to lamin B1 (Fig. 5B), such as RPA1 (replication protein A1) (interaction score = 0.873) and RPA3 (replication protein A3) (interaction score = 0.835). The venn diagram of the above two gene sets showed an intersection consisting of two genes, namely, TMPO and ZWINT (ZW10 interacting kinetochore protein) (Fig. 5C). Then, we carried out KEGG and GO enrichment analysis with the union of the above two gene sets. As shown in Fig. 5D, KEGG analysis revealed that “cell cycle” and “DNA replication” pathways appeared to play important roles in the influence of LMNB1 on the tumorigenesis and development (Fig. 5D). GO term analysis showed that “nuclear division”, “organelle fission”, “mitotic nuclear division” and “chromosome segregation” in biological process (Fig. 5E, F), “chromosomal region” and “spindle” in cellular component (Additional file 1: Fig. S4A, C), “tubulin binding” and “microtubule binding” in molecular function (Additional file 1: Fig. S4B, D) were most involved in the effects of LMNB1 on human tumors. Overall, we hold the opinion that LMNB1 played an important role in tumor growth and cell mitosis.

### LMNB1 was required for DNA homologous recombination repair and contributed to PARPi resistance

**Fig. 6:**
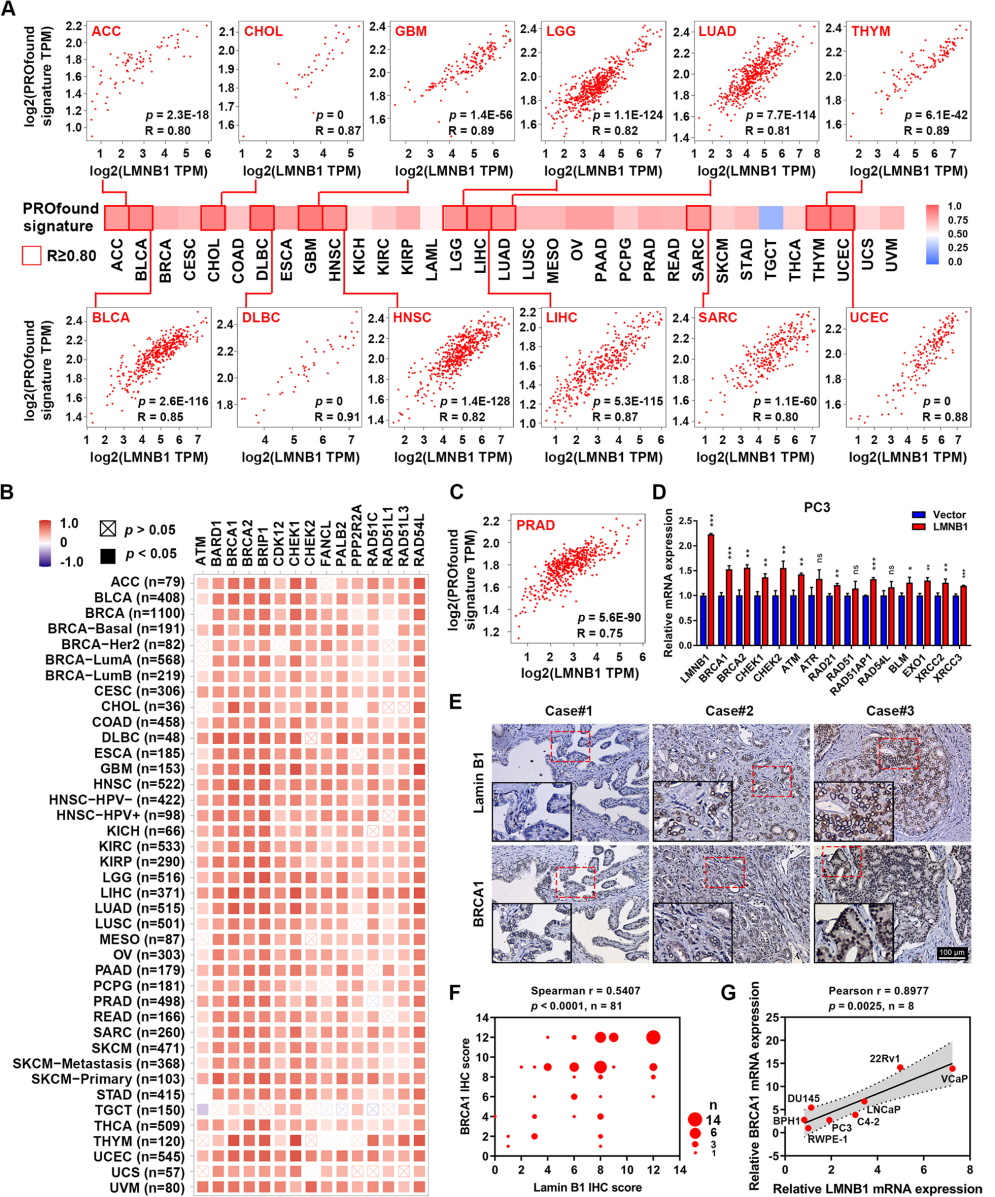
Correlation between LMNB1 expression and DNA homologous recombination repair. **A** The correlation of mRNA expression between LMNB1 gene and PROfound signature across all types of cancer in TCGA was calculated by GEPIA2. The overview was presented in a heatmap while cancers with Pearson correlation coefficient equal to or greater than 0.80 were highlighted with solid line borders and showed in detail. **B** The correlation of mRNA expression between LMNB1 and every single gene of PROfound signature in TCGA datasets was analyzed and visualized by TIMER2.0. **C** The correlation of mRNA expression between LMNB1 gene and PROfound signature in PRAD was investigated with GEPIA2. **D** Relative expression of HRR genes was determined by qRT-PCR in LMNB1-overexpressing and control cells. **E** Representative pictures of lamin B1 and BRCA1 IHC staining in PRAD specimens (n = 81). Scale bars represent 100 μm. **F** The correlation between lamin B1 and BRCA IHC scores in PRAD was evaluated by Spearman correlation analysis. **G** The correlation between LMNB1 and BRCA mRNA expression in prostate cells was examined with Pearson correlation analysis

Interestingly, we found that several genes in the gene aggregate which encodes lamin B1-binding proteins or shows similar expression pattern with LMNB1 were also associated with DNA HRR, such as BRCA1, CHEK1, RAD51, RAD54L and BRIP1 (Fig. 5F, Additional file 1: Fig. S4C, D). To further explore the detailed role of LMNB1 on the DNA HRR, we used GEPIA2 to analyze the correlation between the expression of LMNB1 and a self-defined gene set named as PROfound signature, which consists of 15 HRR-associated genes prespecified by Johann de Bono, et al. in their phase 3 PROfound clinical trials [25]. The heatmap showed that LMNB1 expression had a strong positive correlation with the PROfound signature in the vast majority of TCGA cancer types, and the corresponding scatter plots of the cancer types whose Pearson correlation coefficient was not less than 0.80 were displayed (Fig. 6A). In addition, TIMER2.0 assisted us to show the detail of the correlation between LMNB1 and every single gene in the PROfound signature across all TCGA tumor kinds (Fig. 6B).

PROfound trials revealed that metastatic castration-resistant prostate cancer (CRPC) patients with at least one alteration in the PROfound signature genes would benefit for progression-free survival from olaparib (a selective PARP inhibitor) treatment [25]. Since LMNB1 was revealed to be closely related to the PROfound signature, we speculated that LMNB1 might influence the treatment effects of olaparib. Firstly, in the PRAD cohort of TCGA database, LMNB1 appeared to be strongly correlated with the PROfound signature (R = 0.75, *p* = 5.6E−90) (Fig. 6C). Then we stably overexpressed LMNB1 gene in a CRPC cell line PC3, and qRT-PCR analysis showed that forced expression of LMNB1 increased the mRNA level of BRCA1, BRCA2, CHEK1, CHEK2 and ATM (Fig. 6D). To further validate the relationship between the expression of LMNB1 and HRR genes in PRAD, lamin B1 and BRCA1, which was selected as a representative of HRR proteins were examined in 81 paraffin-embedded prostate cancer tissues by IHC staining (Fig. 6E). As expected, we found that the tumor epithelial areas in the PRAD specimens displaying strong staining of lamin B1 also had heavy signals of BRCA1 (Fig. 6E). Statistically, a positive relationship between lamin B1 and BRCA1 was observed (Spearman r = 0.5407, *p* < 0.0001) (Fig. 6F). In addition, Pearson correlation analysis also indicated that LMNB1 mRNA level was significantly associated with BRCA1 mRNA expression in a panel of prostate cell lines (Pearson r = 0.8977, *p* = 0.0025) (Fig. 6G).

**Fig. 7:**
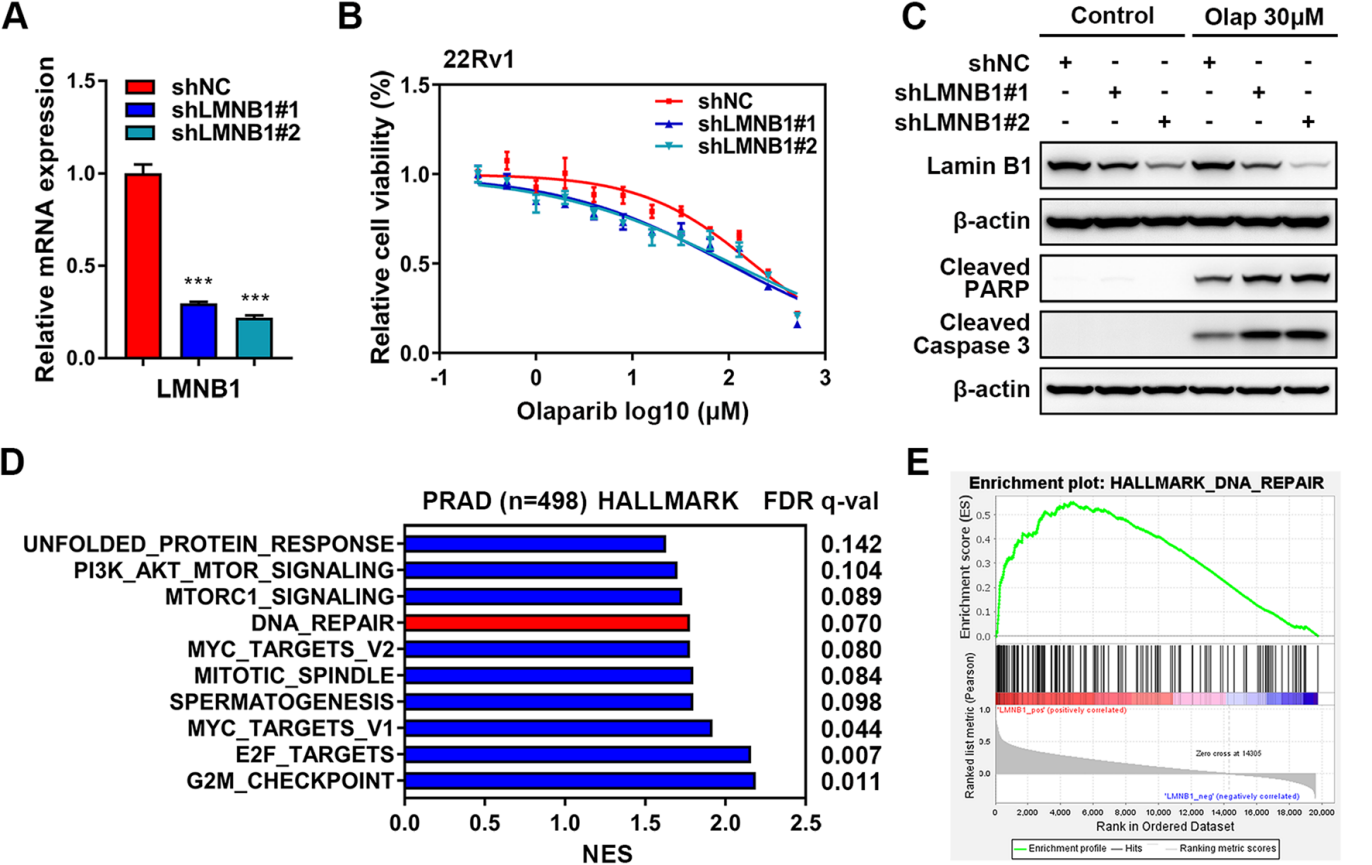
LMNB1 knockdown promoted the antitumor effects of PARPi in PRAD. **A** The knockdown efficiency of specific shRNAs targeting LMNB1 gene in 22Rv1 cells was validated by qRT-PCR. **B** Relative cell viability of LMNB1-deficient 22Rv1 cells exposed to a series of concentrations of olaparib was determined by CCK-8. **C** Western blotting was carried out to detect the levels of cleaved PARP and cleaved caspase3 in LMNB1 knockdown cells in the condition of olaparib treatment. **D** Differentially expressed HALLMARK gene sets with high LMNB1 expression in the TCGA PRAD cohort was screened out by GSEA. NES, normalized enrichment score. **E** The altered genes in the “DNA repair” pathway in the high LMNB1 group compared to low groups from the TCGA PRAD cohort were plotted

In order to investigate the effects of LMNB1 on the PARPi therapy, we stably knocked know LMNB1 in another PRAD cell line 22Rv1, by infecting with two lentiviruses containing specific short hairpin RNAs (shRNAs) targeting different regions of LMNB1 gene (Fig. 7A, Additional file 2: Table S2). The cell viability assay showed that silencing of LMNB1 in 22Rv1 cells resulted in a prominent decreased IC_50_ of olaparib (97.1 μM for shLMNB1#1 and 108.8 μM for shLMNB1#2 versus 177.6 μM for shNC) (Fig. 7B). Western blotting also demonstrated that LMNB1 knockdown caused more serious cell apoptosis characterized by cleaved PARP and cleaved caspase3 in the condition of olaparib incubation (Fig. 7C). Finally, GSEA of PRAD cohort in TCGA database showed that the pathways of “DNA repair” in hallmark gene set (Fig. 7D, E, Additional file 3: Table S3), “homologous recombination”, “mismatch repair”, “nucleotide excision repair”, “base excision repair” in KEGG gene set (Additional file 1: Fig. S5A, B, Additional file 4: Table S4), and “double strand break repair” in GO biological process gene set (Additional file 1: Fig. S5C, D, Additional file 5: Table S5) were positively associated with high LMNB1 expression, indicating that LMNB1 did play a crucial role on DNA repair and PARPi therapy.

## Discussion

LMNB1 is a major constituent of the nuclear lamina and plays important roles in diverse chromatin-associated processes. It has been recognized that duplication of LMNB1 gene or mutation in its promoter leading to increased expression of the encoded protein is a genetic cause of adult-onset autosomal dominant leukodystrophy (ADLD), which is a fatal progressive neurological disease characterized by widespread central nervous system demyelination [2, 44, 45]. The role of LMNB1 in tumor initiation and progression still remain elusive. Based on the data of TCGA, we found that LMNB1 was highly expressed in not only lung adenocarcinoma [11], prostate cancer [12, 13], cervical cancer [14] and liver cancer [16], but also most other human cancer types. Though previous reports indicated that LMNB1 was downregulated in breast cancer by qRT-PCR [17], gastric cancer [18], lung adenocarcinoma and squamous cell carcinoma by IHC staining [19], TCGA analysis indicated LMNB1 expression in the above 4 tumor kinds was upregulated at least in the mRNA level. Besides, proteomic data from CPTAC also demonstrated that lamin B1 expression in the protein level was upregulated in breast cancer and lung adenocarcinoma. Consistent with bioinformatics analysis, a moderate cohort of consecutive KIRC patients in our center also confirmed that LMNB1 was highly expressed in cancerous tissues and positively correlated with cancer aggressiveness. Further research based on multicenter design and relatively large sample size will help to draw a solid conclusion of LMNB1 expression in tumor tissues.

Increasing evidence indicates that LMNB1 is associated with tumor proliferation and metastasis in several types of human cancer, but as far as we know, there is little investigation focusing on the relationship between LMNB1 expression and immune cell infiltration. In this study, we first demonstrated a statistical positive correlation between the mRNA expression of LMNB1 and the infiltration level of CD4+ Th2 cells in all the TCGA tumor types excluding UCS. Th1 and Th2 cells are two distinct subsets of CD4+ T lymphocytes, and Th1/Th2 ratio is in balance under normal conditions [46]. During extracellular pathogen infection, naïve Th cells proliferate and differentiate toward the Th2 subtype, which functions through secreting various effector cytokines, including IL-4, IL-5, IL-6, IL-10, IL-13. Previous studies indicate that Th1/Th2 imbalance has been observed with an elevation of Th2 cells and Th2-released cytokines in several kinds of human cancers [38–43]. Furthermore, patients with Th2 dominant response in TME have poorer prognosis than those with a balance Th1/Th2 infiltration [43, 47]. Therefore, development of new strategies targeting LMNB1 or Th2 cells may help to redress Th1/Th2 imbalance and improve the prognosis of tumor patients with high LMNB1 expression.

In our study, KEGG enrichment analysis using LMNB1-related partners based on TCGA datasets indicated that “cell cycle” and “DNA replication” are concerned in the effects of LMNB1 on tumor pathogenesis. These findings have been evidenced in previous studies that lamin B1 is a key component of the nuclear lamina providing a framework for the nuclear envelope and is required for the initiation phase or the elongation phase of DNA replication [48]. Butin-Israeli et al. found that LMNB1 knockdown by shRNA in cancer cells slowed the cell cycle with the S phase delay, which was accompanied by the stalling and collapse of replication forks [7]. Furthermore, the replication fork collapse resulted in the increase of double-strand DNA breaks (DSB), which were inefficiently repaired in LMNB1 deficient cells [7]. Their results suggested that the maintain of LMNB1 expression was also required for chromatin stability and DNA repair. Consistently, in the LMNB1-related cellular process in cancer cells including “nuclear division”, “organelle fission”, “mitotic nuclear division” and “chromosome segregation”, which were enriched by GO pathway analysis using TCGA data, we found several HRR genes such as BRCA1, CHEK1, RAD51, RAD54L and BRIP1 were implicated. Further investigation confirmed a strong positive relevance between LMNB1 expression and the 15 HRR genes in the PROfound trial in almost all TCGA cancer types. Practically, IHC staining of prostate cancer tissues in our center and qRT-PCR analysis of prostate cell lines also showed a close correlation between LMNB1 and BRCA1 expression. Forced expression of LMNB1 in prostate cancer cells increased the mRNA level of several HRR genes, especially BRCA1. These findings were inconsistent with Butin-Israeli V’s research that BRCA1 expression was increased in LMNB1 knockdown cells by shRNA in osteosarcoma cell line U-2-OS and colorectal carcinoma cell line HCT116 [7]. One possible explanation for the inconsistent findings may be that the mechanism of LMNB1 on regulating BRCA1 is distinct in different tumor types. On the other hand, the off-target effects in previous study could not be completely ruled out since only one shRNA was used to silence LMNB1 expression [7].

Tumor cells with deficiency of HRR genes, especially BRCA1 and BRCA2, are sensitive to PARPi through the mechanism of synthetic lethality [49, 50]. Since LMNB1 is tightly correlated with HRR genes and plays an important role in DNA repair, we speculate that targeting LMNB1 could synergistically promote the effects of PARPi on cancer cells. Cell viability assay and western blotting experiment validate our thoughts, and these findings suggest that combination therapy of LMNB1 knockdown and PARPi could improve the prognosis of cancer patients without loss-of-function alteration of HRR genes.

## Conclusions

In conclusion, our pan-cancer analysis provides a comprehensive overview of the oncogenic roles of LMNB1 in human cancers. Overexpression of LMNB1 generally predicted poor prognosis for cancer patients and suggested high level of CD4+ Th2 cell infiltration. Moreover, LMNB1 was universally correlated with HRR genes, which may serve as a potential therapeutic target to enhance the antitumor effect effects of PARPi in human cancers.

## Supplementary Information

**Additional file 1: Figure S1.** LMNB1 expression in human tissues, cells and plasma. **Figure S2.** Correlation between LMNB1 mRNA expression and CD4+ Th2 infiltration in TCGA cancers. **Figure S3.** Relevance between mRNA expression of LMNB1 and its correlated genes. **Figure S4.** GO term enrichment analysis of LMNB1-related genes across all TCGA cancers. **Figure S5.** GSEA of LMNB1 expression in the PARD cohort of TCGA datasets.

**Additional file 2: Table S1.** Primer sequences used in qRT-PCR. **Table S2.** shRNA sequence for gene knockdown.

**Additional file 3: Table S3.** GESA with HALLMARK gene sets of PRAD cohort in TCGA.

**Additional file 4: Table S4.** GESA with KEGG gene sets of PRAD cohort in TCGA.

**Additional file 5: Table S5.** GESA with GO_BP gene sets of PRAD cohort in TCGA.

## Data Availability

The public datasets involved in this study are available in online repositories. Original contributions presented in this study are included in the manuscript or additional files. Further inquiries can be directed to the corresponding author.

## Abbreviations

TCGA: The Cancer Genome Atlas
TIMER2.0: Tumor Immune Estimation Resource version 2
GEPIA2: Gene Expression Profiling Interactive Analysis version 2
GEO: Gene Expression Omnibus
CPTAC: Clinical Proteomic Tumor Analysis Consortium
PARP: Poly ADP-ribose polymerase
PARPi: PARP inhibitor
CCK-8: Cell Counting Kit 8
qRT-PCR: Quantitative real-time PCR
IHC: Immunohistochemistry
HPA: The Human Protein Atlas
NX: Normalized expression
GTEx: The Genotype-Tissue Expression
FANTOM5: The Functional Annotation of Mammalian Genomes 5
OS: Overall survival
DFS: Disease free survival
KEGG: Kyoto encyclopedia of genes and genomes
GO: Gene ontology
DAVID: Database for Annotation, Visualization, and Integrated Discovery
GSEA: Gene Set Enrichment Analysis
MSigDB: The Molecular Signatures Database
BLCA: Bladder Urothelial Carcinoma
BRCA: Breast invasive carcinoma
CESC: Cervical squamous cell carcinoma and endocervical adenocarcinoma
CHOL: Cholangio carcinoma
COAD: Colon adenocarcinoma
ESCA: Esophageal carcinoma
GBM: Glioblastoma multiforme
HNSC: Head and neck squamous cell carcinoma
KIRC: Kidney renal clear cell carcinoma
KIRP: Kidney renal papillary cell carcinoma
LIHC: Liver hepatocellular carcinoma
LUAD: Lung adenocarcinoma
LUSC: Lung squamous cell carcinoma
PCPG: Pheochromocytoma and Paraganglioma
PRAD: Prostate adenocarcinoma
READ: Rectum adenocarcinoma
STAD: Stomach adenocarcinoma
THCA: Thyroid carcinoma
UCEC: Uterine Corpus Endometrial Carcinoma
KICH: Kidney Chromophobe
SKCM: Skin Cutaneous Melanoma
OV: Ovarian serous cystadenocarcinoma
ACC: Adrenocortical carcinoma
TGCT: Testicular Germ Cell Tumors
LGG: Brain Lower Grade Glioma
MESO: Mesothelioma
SARC: Sarcoma
THYM: Thymoma
UVM: Uveal Melanoma
UCS: Uterine Carcinosarcoma
TME: The tumor microenvironment
HRR: Homologous recombination repair
CRPC: Castration-resistant prostate cancer
shRNAs: Short hairpin RNAs
ADLD: Adult-onset autosomal dominant leukodystrophy
DSB: Double-strand DNA breaks

## References

[1] Dittmer TA, Misteli T (2011). The lamin protein family. Genome Biol.

[2] Padiath QS (2019). Autosomal dominant leukodystrophy: a disease of the nuclear lamina. Front Cell Dev Biol.

[3] Worman HJ (2012). Nuclear lamins and laminopathies. J Pathol.

[4] Bukata L, Parker SL, D'Angelo MA (2013). Nuclear pore complexes in the maintenance of genome integrity. Curr Opin Cell Biol.

[5] Dechat T, Adam SA, Taimen P, Shimi T, Goldman RD (2010). Nuclear lamins. Cold Spring Harbor Perspect Biol.

[6] Malhas AN, Lee CF, Vaux DJ (2009). Lamin B1 controls oxidative stress responses via Oct-1. J Cell Biol.

[7] Butin-Israeli V, Adam SA, Jain N, Otte GL, Neems D, Wiesmüller L (2015). Role of lamin b1 in chromatin instability. Mol Cell Biol.

[8] Liu NA, Sun J, Kono K, Horikoshi Y, Ikura T, Tong X (2015). Regulation of homologous recombinational repair by lamin B1 in radiation-induced DNA damage. FASEB J.

[9] Tang CW, Maya-Mendoza A, Martin C, Zeng K, Chen S, Feret D (2008). The integrity of a lamin-B1-dependent nucleoskeleton is a fundamental determinant of RNA synthesis in human cells. J Cell Sci.

[10] Dobrzynska A, Gonzalo S, Shanahan C, Askjaer P (2016). The nuclear lamina in health and disease. Nucleus (Austin, Tex).

[11] Li W, Li X, Li X, Li M, Yang P, Wang X (2020). Lamin B1 overexpresses in lung adenocarcinoma and promotes proliferation in lung cancer cells via AKT pathway. Onco Targets Ther.

[12] Luo F, Han J, Chen Y, Yang K, Zhang Z, Li J (2021). Lamin B1 promotes tumor progression and metastasis in primary prostate cancer patients. Future Oncol (Lond, Engl).

[13] Song ZY, Chao F, Zhuo Z, Ma Z, Li W, Chen G (2019). Identification of hub genes in prostate cancer using robust rank aggregation and weighted gene co-expression network analysis. Aging.

[14] Yang Z, Sun Q, Guo J, Wang S, Song G, Liu W (2019). GRSF1-mediated MIR-G-1 promotes malignant behavior and nuclear autophagy by directly upregulating TMED5 and LMNB1 in cervical cancer cells. Autophagy.

[15] Li L, Du Y, Kong X, Li Z, Jia Z, Cui J (2013). Lamin B1 is a novel therapeutic target of betulinic acid in pancreatic cancer. Clin Cancer Res.

[16] Sun S, Xu MZ, Poon RT, Day PJ, Luk JM (2010). Circulating Lamin B1 (LMNB1) biomarker detects early stages of liver cancer in patients. J Proteome Res.

[17] Wazir U, Ahmed MH, Bridger JM, Harvey A, Jiang WG, Sharma AK (2013). The clinicopathological significance of lamin A/C, lamin B1 and lamin B receptor mRNA expression in human breast cancer. Cell Mol Biol Lett.

[18] Yu ZY, Jiang XY, Zhao RR, Luo CJ, Ren YX, Ma ZJ (2020). Lamin B1 deficiency promotes malignancy and predicts poor prognosis in gastric cancer. Neoplasma.

[19] Jia Y, Vong JS, Asafova A, Garvalov BK, Caputo L, Cordero J (2019). Lamin B1 loss promotes lung cancer development and metastasis by epigenetic derepression of RET. J Exp Med.

[20] Prieto J, Melero I, Sangro B (2015). Immunological landscape and immunotherapy of hepatocellular carcinoma. Nat Rev Gastroenterol Hepatol.

[21] Krishnamoorthy M, Gerhardt L, Maleki VS (2021). Immunosuppressive effects of myeloid-derived suppressor cells in cancer and immunotherapy. Cells.

[22] Costa A, Kieffer Y, Scholer-Dahirel A, Pelon F, Bourachot B, Cardon M (2018). Fibroblast heterogeneity and immunosuppressive environment in human breast cancer. Cancer Cell.

[23] Groth C, Hu X, Weber R, Fleming V, Altevogt P, Utikal J (2019). Immunosuppression mediated by myeloid-derived suppressor cells (MDSCs) during tumour progression. Br J Cancer.

[24] Lv D, Wu X, Wang M, Chen W, Yang S, Liu Y (2021). Functional assessment of four novel immune-related biomarkers in the pathogenesis of clear cell renal cell carcinoma. Front Cell Dev Biol.

[25] de Bono J, Mateo J, Fizazi K, Saad F, Shore N, Sandhu S (2020). Olaparib for metastatic castration-resistant prostate cancer. N Engl J Med.

[26] Golan T, Hammel P, Reni M, Van Cutsem E, Macarulla T, Hall MJ (2019). Maintenance olaparib for germline BRCA-mutated metastatic pancreatic cancer. N Engl J Med.

[27] Qin H, Yang Y, Jiang B, Pan C, Chen W, Diao W (2021). SOX9 in prostate cancer is upregulated by cancer-associated fibroblasts to promote tumor progression through HGF/c-Met-FRA1 signaling. The FEBS journal..

[28] Li T, Fu J, Zeng Z, Cohen D, Li J, Chen Q (2020). TIMER2.0 for analysis of tumor-infiltrating immune cells. Nucleic Acids Res.

[29] Li T, Fan J, Wang B, Traugh N, Chen Q, Liu JS (2017). TIMER: a web server for comprehensive analysis of tumor-infiltrating immune cells. Cancer Res.

[30] Li B, Severson E, Pignon JC, Zhao H, Li T, Novak J (2016). Comprehensive analyses of tumor immunity: implications for cancer immunotherapy. Genome Biol.

[31] Tang Z, Kang B, Li C, Chen T, Zhang Z (2019). GEPIA2: an enhanced web server for large-scale expression profiling and interactive analysis. Nucleic Acids Res.

[32] Chandrashekar DS, Bashel B, Balasubramanya SAH, Creighton CJ, Ponce-Rodriguez I, Chakravarthi B (2017). UALCAN: a portal for facilitating tumor subgroup gene expression and survival analyses. Neoplasia (New York, NY).

[33] Chen F, Chandrashekar DS, Varambally S, Creighton CJ (2019). Pan-cancer molecular subtypes revealed by mass-spectrometry-based proteomic characterization of more than 500 human cancers. Nat Commun.

[34] Mootha VK, Lindgren CM, Eriksson KF, Subramanian A, Sihag S, Lehar J (2003). PGC-1alpha-responsive genes involved in oxidative phosphorylation are coordinately downregulated in human diabetes. Nat Genet.

[35] Subramanian A, Tamayo P, Mootha VK, Mukherjee S, Ebert BL, Gillette MA (2005). Gene set enrichment analysis: a knowledge-based approach for interpreting genome-wide expression profiles. Proc Natl Acad Sci USA.

[36] Kirtane K, Elmariah H, Chung CH, Abate-Daga D (2021). Adoptive cellular therapy in solid tumor malignancies: review of the literature and challenges ahead. J Immunother Cancer.

[37] Hiltbrunner S, Mannarino L, Kirschner MB, Opitz I, Rigutto A, Laure A (2021). Tumor immune microenvironment and genetic alterations in mesothelioma. Front Oncol.

[38] DeNardo DG, Barreto JB, Andreu P, Vasquez L, Tawfik D, Kolhatkar N (2009). CD4(+) T cells regulate pulmonary metastasis of mammary carcinomas by enhancing protumor properties of macrophages. Cancer Cell.

[39] Mahata B, Zhang X, Kolodziejczyk AA, Proserpio V, Haim-Vilmovsky L, Taylor AE (2014). Single-cell RNA sequencing reveals T helper cells synthesizing steroids de novo to contribute to immune homeostasis. Cell Rep.

[40] Maier B, Leader AM, Chen ST, Tung N, Chang C, LeBerichel J (2020). A conserved dendritic-cell regulatory program limits antitumour immunity. Nature.

[41] Liu Z, Zhou Q, Wang Z, Zhang H, Zeng H, Huang Q (2020). Intratumoral TIGIT(+) CD8(+) T-cell infiltration determines poor prognosis and immune evasion in patients with muscle-invasive bladder cancer. J Immunother Cancer.

[42] Zhao X, Liu J, Ge S, Chen C, Li S, Wu X (2019). Saikosaponin A inhibits breast cancer by regulating Th1/Th2 balance. Front Pharmacol.

[43] De Monte L, Reni M, Tassi E, Clavenna D, Papa I, Recalde H (2011). Intratumor T helper type 2 cell infiltrate correlates with cancer-associated fibroblast thymic stromal lymphopoietin production and reduced survival in pancreatic cancer. J Exp Med.

[44] Dai Y, Ma Y, Li S, Banerjee S, Liang S, Liu Q (2017). An LMNB1 duplication caused adult-onset autosomal dominant leukodystrophy in chinese family: clinical manifestations, neuroradiology and genetic diagnosis. Front Mol Neurosci.

[45] Padiath QS, Saigoh K, Schiffmann R, Asahara H, Yamada T, Koeppen A (2006). Lamin B1 duplications cause autosomal dominant leukodystrophy. Nat Genet.

[46] Hirahara K, Nakayama T (2016). CD4+ T-cell subsets in inflammatory diseases: beyond the Th1/Th2 paradigm. Int Immunol.

[47] Ubukata H, Motohashi G, Tabuchi T, Nagata H, Konishi S, Tabuchi T (2010). Evaluations of interferon-γ/interleukin-4 ratio and neutrophil/lymphocyte ratio as prognostic indicators in gastric cancer patients. J Surg Oncol.

[48] Hutchison CJ (2014). B-type lamins in health and disease. Semin Cell Dev Biol.

[49] Lord CJ, Ashworth A (2017). PARP inhibitors: synthetic lethality in the clinic. Science (New York, NY).

[50] Li H, Liu ZY, Wu N, Chen YC, Cheng Q, Wang J (2020). PARP inhibitor resistance: the underlying mechanisms and clinical implications. Mol Cancer.

