# Supplementary material for: Pan-cancer analysis identifies LMNB1 as a target to redress Th1/Th2 imbalance and enhance PARP inhibitor response in human cancers
Source: Cancer Cell Int. 2022 Mar 3;22:101. doi: 10.1186/s12935-022-02467-4 (PMC8896121; doi:10.1186/s12935-022-02467-4)
Supplement: Supplementary file 1 — Additional file 1: Figure S1. LMNB1 expression in human tissues, cells and plasma. Figure S2. Correlation between LMNB1 mRNA expression and CD4+ Th2 infiltration in TCGA cancers. Figure S3. Relevance between mRNA expression of LMNB1 and its correlated genes. Figure S4. GO term enrichment analysis of LMNB1-related genes across all TCGA cancers. Figure S5. GSEA of LMNB1 expression in the PARD cohort of TCGA datasets. [file 12935_2022_2467_MOESM1_ESM.docx]

**Additional figures and legends**


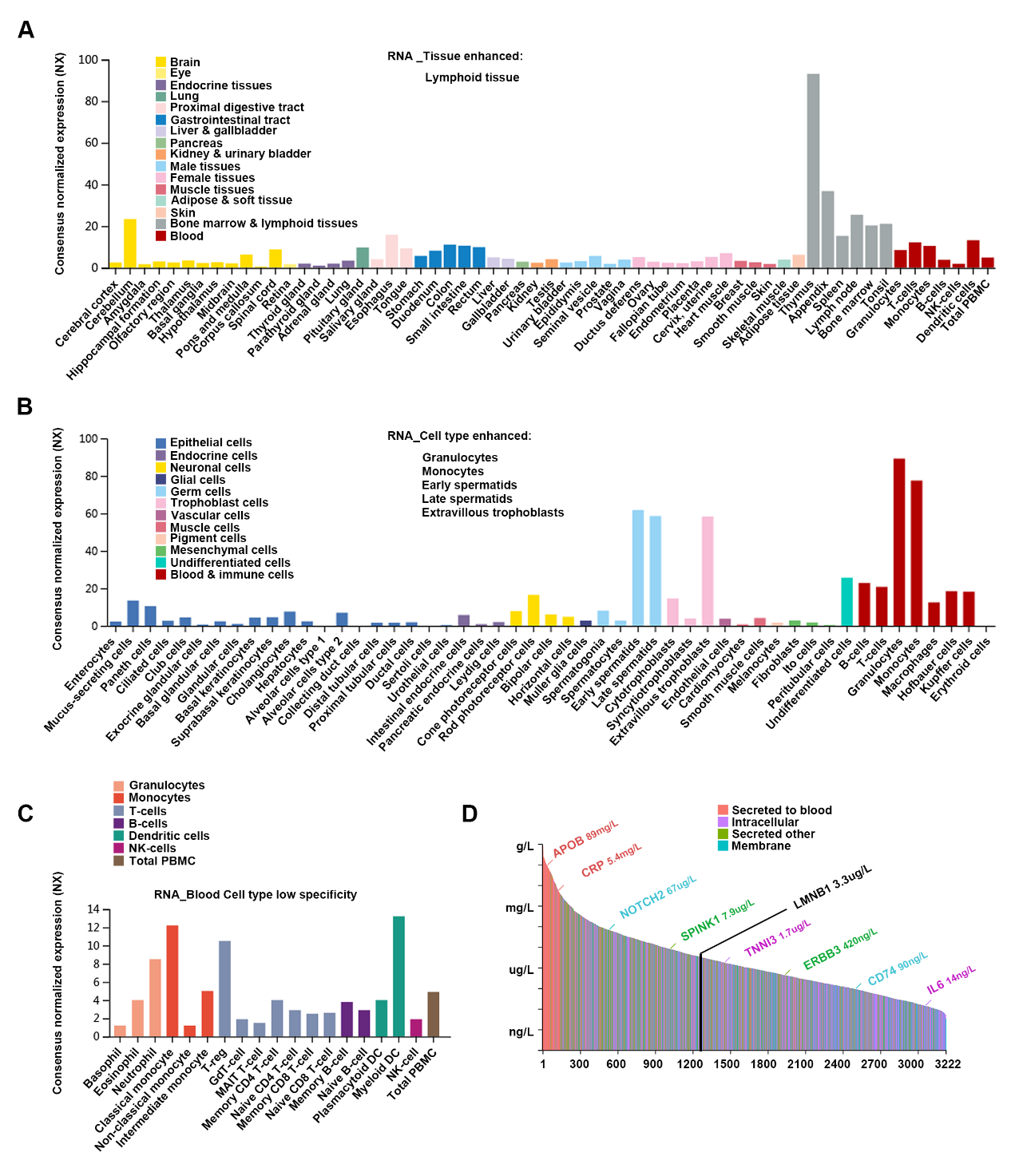


**Figure S1. LMNB1 expression in human tissues, cells and plasma.**

**(A)** NX levels for 55 tissue types and 6 blood cell types, acquired by combining the data from HPA, GTEx and FANTOM5, were visualized in a column diagram. **(B)** NX levels for 51 single cell types were analyzed by respectively retrieved from the datasets of Single Cell Expression Atlas, the Human Cell Atlas, the European Genome-phenome Archive and GEO. **(C)** NX levels for 19 blood cell types were determined using the consensus data of HPA, Monaco et al and Schmiedel et al. **(D)** The Lamin B1 protein concentration in human blood based on mass spectrometry was also displayed.


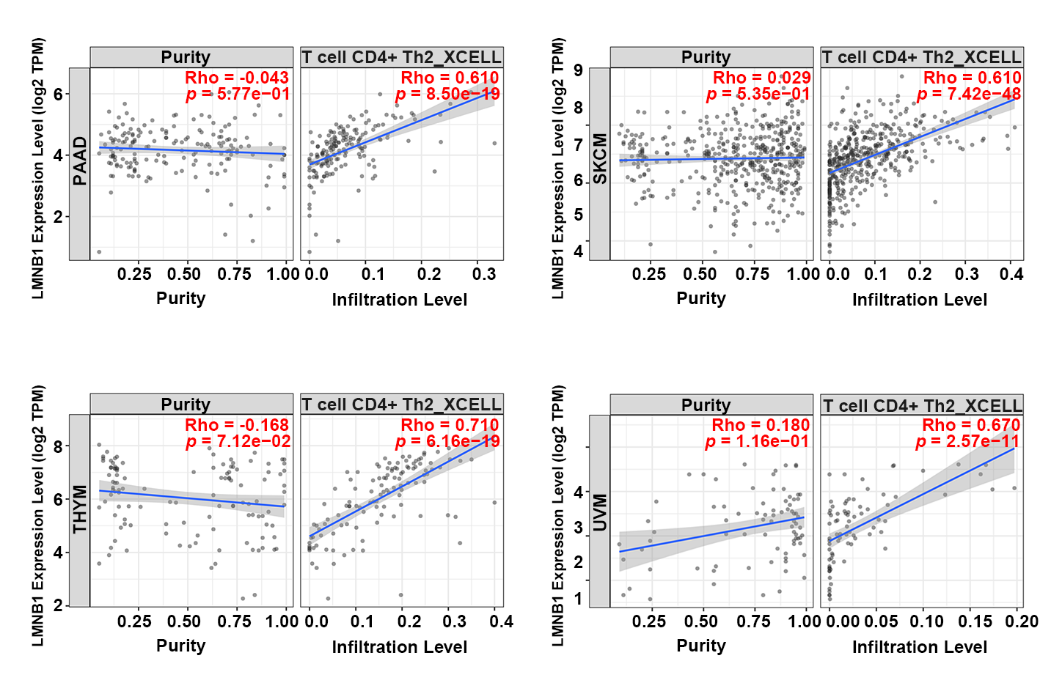


**Figure S2. Correlation between LMNB1 mRNA expression and CD4+ Th2 infiltration in TCGA cancers.**

The scatter plots of cancers with highest purity-adjusted Spearman’s rho coefficient were presented with *p* values.


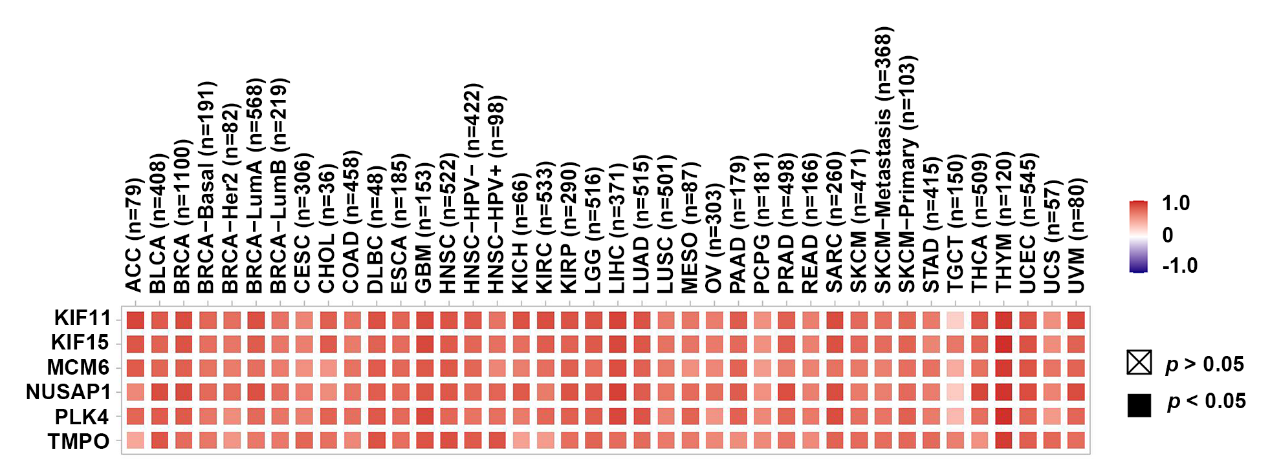


**Figure S3. Relevance between mRNA expression of LMNB1 and its correlated genes.**

The heatmap showed the detail of the correlation between LMNB1 and KIF11, KIF15, MCM6, NUSAP1, PLK4 and TMPO across all the TCGA tumor types.


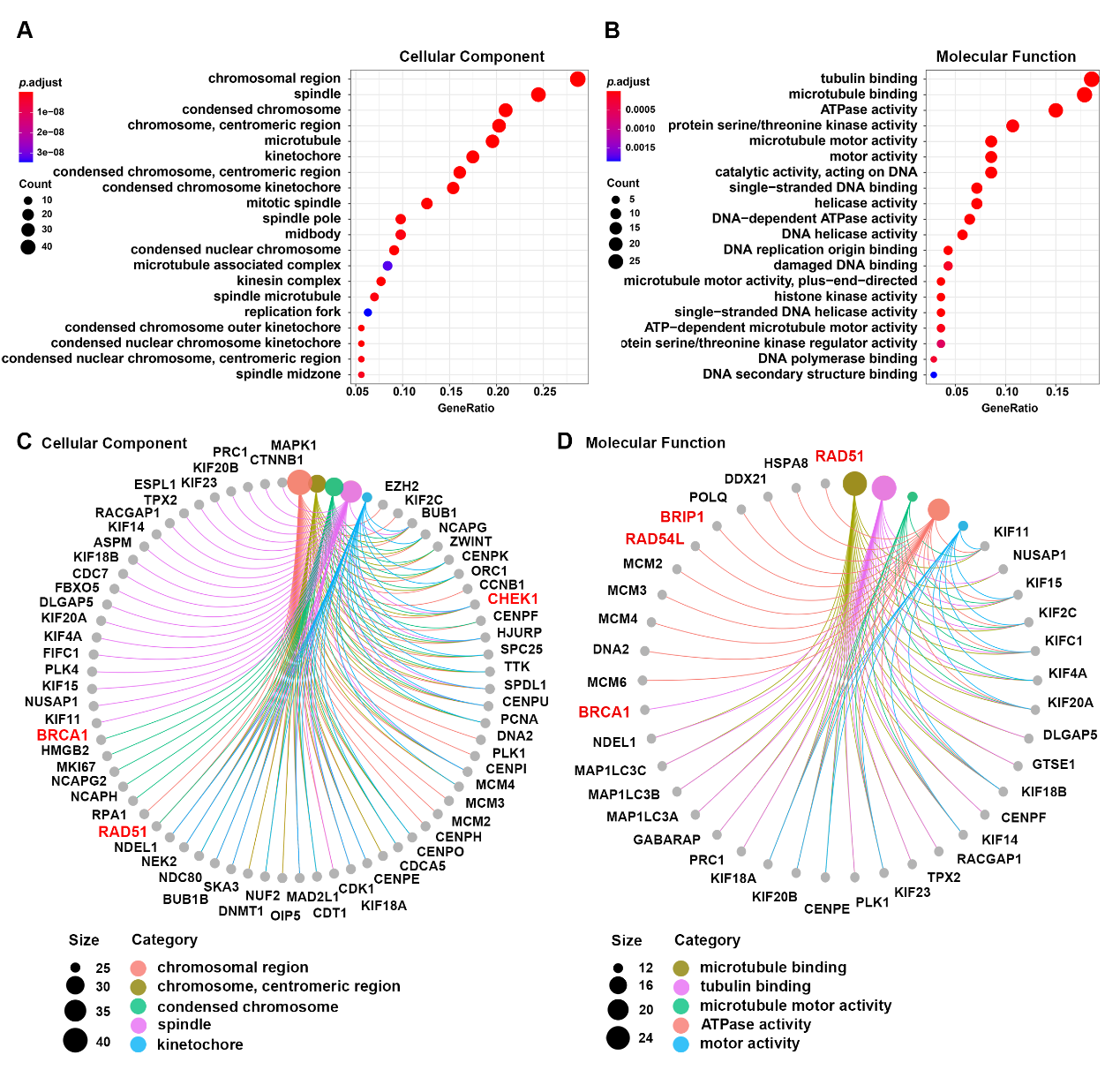


**Figure S4. GO term enrichment analysis of LMNB1-related genes across all TCGA cancers.**

**(A, B)** GO enrichment analysis of cellular component (A) and molecular function (B) was conducted based on LMNB1-correlated genes and lamin B1-interected proteins in TCGA. **(C, D)** Genes implicated in the most correlated GO terms were showed in detail.


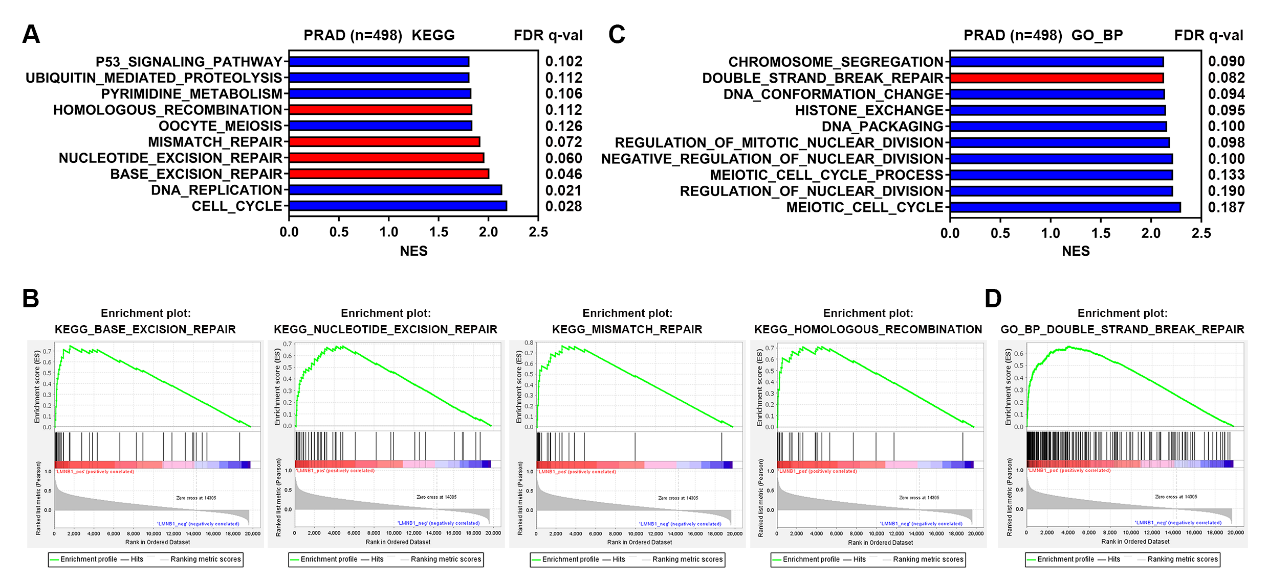


**Figure S5. GSEA of LMNB1 expression in the PARD cohort of TCGA datasets.**

**(A, C)** Gene set enrichment of KEGG (A) and GO biological process (C) with LMNB1 upregulation was performed using the data of the TCGA PRAD cohort. **(B, D)** Involved genes in the enriched pathways related to DNA repair were plotted.
