# Supplementary material for: Pan-cancer analysis identifies LMNB1 as a target to redress Th1/Th2 imbalance and enhance PARP inhibitor response in human cancers
Source: Cancer Cell Int. 2022 Mar 3;22:101. doi: 10.1186/s12935-022-02467-4 (PMC8896121; doi:10.1186/s12935-022-02467-4)
Supplement: Supplementary file 2 — Additional file 2: Table S1. Primer sequences used in qRT-PCR. Table S2. shRNA sequence for gene knockdown. [file 12935_2022_2467_MOESM2_ESM.docx]

**Table S1: Primer sequences used in qRT-PCR.**

| **Genes** |  | **Sequence** |
| --- | --- | --- |
| LMNB1 | Forward (5’-3’) | GCTCTTGCTACTGCACTTGG |
|  | Reverse (5’-3’) | GCGCGTTTCATGCTTCCTTC |
| BRCA1 | Forward (5’-3’) | TGCGGGAGGAAAATGGGTAG |
|  | Reverse (5’-3’) | TCTTGCTCGCTTTGGACCTT |
| BRCA2 | Forward (5’-3’) | GCCAAGTCATGCCACACATTC |
|  | Reverse (5’-3’) | TGCCATCTGGAGTGCTTTTTG |
| CHEK1 | Forward (5’-3’) | TCAAGAAAGGGGCAAAAAGGC |
|  | Reverse (5’-3’) | TCAATGTATGAGGGGCTGGT |
| CHEK2 | Forward (5’-3’) | ACAGCTCTACCCCAGGTTCT |
|  | Reverse (5’-3’) | ACCACGGAGTTCACAACACA |
| ATM | Forward (5’-3’) | CTGCTTATCTGCTGCCGTCAA |
|  | Reverse (5’-3’) | GGCTTGTGTTGAGGCTGATAC |
| ATR | Forward (5’-3’) | CCACACTGAGAACTGGCCTTC |
|  | Reverse (5’-3’) | CTTGCACAGCATCCCTGTTT |
| RAD21 | Forward (5’-3’) | ACCGCCCACCAAGAAATTGA |
|  | Reverse (5’-3’) | CAGCGTGTAAAGAGCTTCAGT |
| RAD51 | Forward (5’-3’) | AGTGGCTGAGAGGTATGGTCT |
|  | Reverse (5’-3’) | GTCTGTTCTGTAAAGGGCGGT |
| RAD51AP1 | Forward (5’-3’) | TCTGGAAGGCAGTGATGGTGA |
|  | Reverse (5’-3’) | GGAGCAGAGTCCACCGAAGT |
| RAD54L | Forward (5’-3’) | TCCATCGAGCCCTGACTTTGT |
|  | Reverse (5’-3’) | TGCCTTCTTGTGGCTCTGACG |
| BLM | Forward (5’-3’) | ACAGCTTTTGGCCTACTTTGG |
|  | Reverse (5’-3’) | ACATCGTCAGTCACATCTCTTGT |
| EXO1 | Forward (5’-3’) | TGGGATCAAGCCTATTCTCGT |
|  | Reverse (5’-3’) | GCTTCCGAGACTTTCCCCTC |
| XRCC2 | Forward (5’-3’) | GGCGATGTGTAGTGCCTTCC |
|  | Reverse (5’-3’) | TCCAGGCCACCTTCTGATTTG |
| CRCC3 | Forward (5’-3’) | CCAGCCCACCGACAAAATG |
|  | Reverse (5’-3’) | TTCCCCGCAAGTGTAAGGAG |

**Table S2: shRNA sequence for gene knockdown**

| **Name** | **Target sequence (**5’-3’**)** | **Loop** |
| --- | --- | --- |
| shNC | gcTTCTCCGAACGTGTCACGT | CTCGAG |
| shLMNB1#1 | gcATGAGAATTGAGAGCCTTT | CTCGAG |
| shLMNB1#2 | gcTCAAAGAAGTACAGTCTTT | CTCGAG |
